# ncRI: a manually curated database for experimentally validated non-coding RNAs in inflammation

**DOI:** 10.1186/s12864-020-06794-6

**Published:** 2020-06-01

**Authors:** Shuyuan Wang, Shunheng Zhou, Haizhou Liu, Qianqian Meng, Xueyan Ma, Hui Liu, Lihong Wang, Wei Jiang

**Affiliations:** 1 College of Bioinformatics Science and Technology, Harbin Medical University, Harbin, 150081 China; 2 College of Automation Engineering, Nanjing University of Aeronautics and Astronautics, Nanjing, 211106 China; 3 Department of Pathophysiology, School of Medicine, Southeast University, Nanjing, 210009 China

**Keywords:** Inflammatory process, Inflammatory disease, Immune factor, Non-coding RNA, Database

## Abstract

**Background:**

Inflammation has been considered to be central to the onset, progression, and outcome of infectious diseases, especially as one of the hallmarks of cancer. Non-coding RNAs (ncRNAs), such as miRNAs and lncRNAs, have emerged as vital regulators in control of immune and inflammatory processes, and also play important roles in the inflammatory disease and immunotherapy.

**Results:**

In this study, we presented a database ncRI, which documented experimentally verified ncRNAs in inflammatory diseases, from published articles. Each entry contained the detailed information about ncRNA name, inflammatory diseases, mechanism, experimental techniques (e.g., microarray, RNA-seq, qRT-PCR), experimental samples (cell line and/or tissue), expression patterns of ncRNA (up-regulated or down-regulated), reference information (PubMed ID, year of publication, title of paper) and so on. Collectively, ncRI recorded 11,166 entries that include 1976 miRNAs, 1377 lncRNAs and 107 other ncRNAs across 3 species (human, mouse, and rat) from more than 2000 articles. All these data are free for users to search, browse and download.

**Conclusion:**

In summary, the presented database ncRI provides a relatively comprehensive credible repository about ncRNAs and their roles in inflammatory diseases, and will be helpful for research on immunotherapy. The ncRI is now freely available to all users at http://www.jianglab.cn/ncRI/.

## Background

Inflammation is a series of the complicated organic response of body to harmful stimulus, and deregulated inflammation may cause damage to organic tissues [1]. Recently, inflammation is considered to be one of the enabling hallmarks of cancer, and it has been estimated that more than 20% of cancers are caused by chronic inflammation [1, 2]. The inflammatory mediators, such as cytokines and chemokines, can regulate the behavior of the immune system and are involved in the immunotherapy [1, 3]. For example, IL-2 is proved to enhance anti-tumor activity and has been used in the treatment of malignant melanoma and renal cell carcinoma [4]. With the discovery of non-coding RNAs (ncRNAs), such as miRNAs and lncRNAs, a further level of control in immunity and inflammatory processes arouses interests in inflammation related research. NcRNAs have been demonstrated to play important roles in immune system and represent novel potential targets for immunotherapy [5, 6]. For example, Murugaiyan et al. demonstrated that miR-155 elicited susceptibility to experimental autoimmune encephalomyelitis (EAE), and anti-miR-155 treatment could alleviate clinical severity of EAE [7]. The lncRNA MALAT1 was verified to up-regulate glucose causal inflammatory factors IL-6 and TNF-α, leading to the potential development of therapeutics targeting MALAT1 for diabetes related vascular complications [8].

However, the knowledge about inflammatory disease related ncRNAs scattered in a large amount of literature without a systematic recording. Lu et al. provided a curated database GAAD to record genes and autoimmune diseases association [9] and Prabahar et al. presented an integrated human immune disease associated miRNAs database ImmunemiR to provide a repository for immune related disease and miRNA associations. Despite some progress, more laborious work remain to be done to gather the immune and inflammatory disease related factors [10]. Especially, a global view of the inflammatory disease associated ncRNAs will help to characterize the ncRNAs roles in inflammation and inspire disease therapy.

To fulfill this purpose, we presented a database ncRI which collected experimentally validated ncRNAs in inflammatory disease from published papers. The related publications were manually curated from the PubMed database. Then, the information about ncRNAs and their roles in inflammatory disease were retrieved. The current version of ncRI documents 11,166 manually curated entries across 3 species (human, mouse, rat), and each entry in this database encompass comprehensive information about the association. We hope that this elaborate database specially designed for inflammatory disease associated ncRNAs could be helpful for research on immunotherapy.

## Construction and content

We explored experimentally validated ncRNAs in inflammatory disease from PubMed database by using a series of keyword search, such as “miRNA and inflammation”, “microRNA and immune”, “lncRNA and inflammation”, “non-coding RNA and inflammation” and so on. Approximately 9000 papers that were published before January 2020 were obtained. After manually screening, we got 11,166 entries that include 1976 miRNAs, 1377 lncRNAs and 107 other ncRNAs across 3 species (human, mouse, rat) from more than 2000 papers. Each entry contained ncRNA name, description of ncRNAs involved in inflammatory diseases, mechanism, experimental methods (e.g., RNA-seq, microarray, qRT-PCR), expression direction of ncRNAs (down-regulated or up-regulated), experimental samples (tissues or/and cell lines), reference information (PubMed ID, the publication year, the paper title) and species. We further unified miRNA name by miRBase version 21.0, and obtained miRBase accession ID for miRNA and ensembl ID for lncRNAs and other ncRNAs. In addition, we provided a tree view of the curated inflammatory diseases based on the MeSH (Medical Subject Headings) Diseases Category, which will help systematically and normatively study the associations between ncRNAs and inflammation.

It is worth noting that the experimental methods for detecting the inflammatory disease related ncRNAs involved various experimental techniques, which could be summarized as high throughput methods, such as microarray and RNA-seq, and low throughput methods, such as qRT-PCR. The high throughput methods would introduce more potential associated ncRNAs, but they would also bring about false-positive associations. The low throughput methods presented more reliable relationships, but relatively few associations were provided. To balance the abundance and credibility of the curated associations, we kept both experimental data in our database. In total, 7400 entries and 3766 entries were obtained through high throughput experiment and low throughput experiment, respectively. Finally, we developed ncRI by using JSP, tomcat 8.5.5 and MySQL, and ncRI runs under CentOS system.

## Utility and discussion

**Fig. 1:**
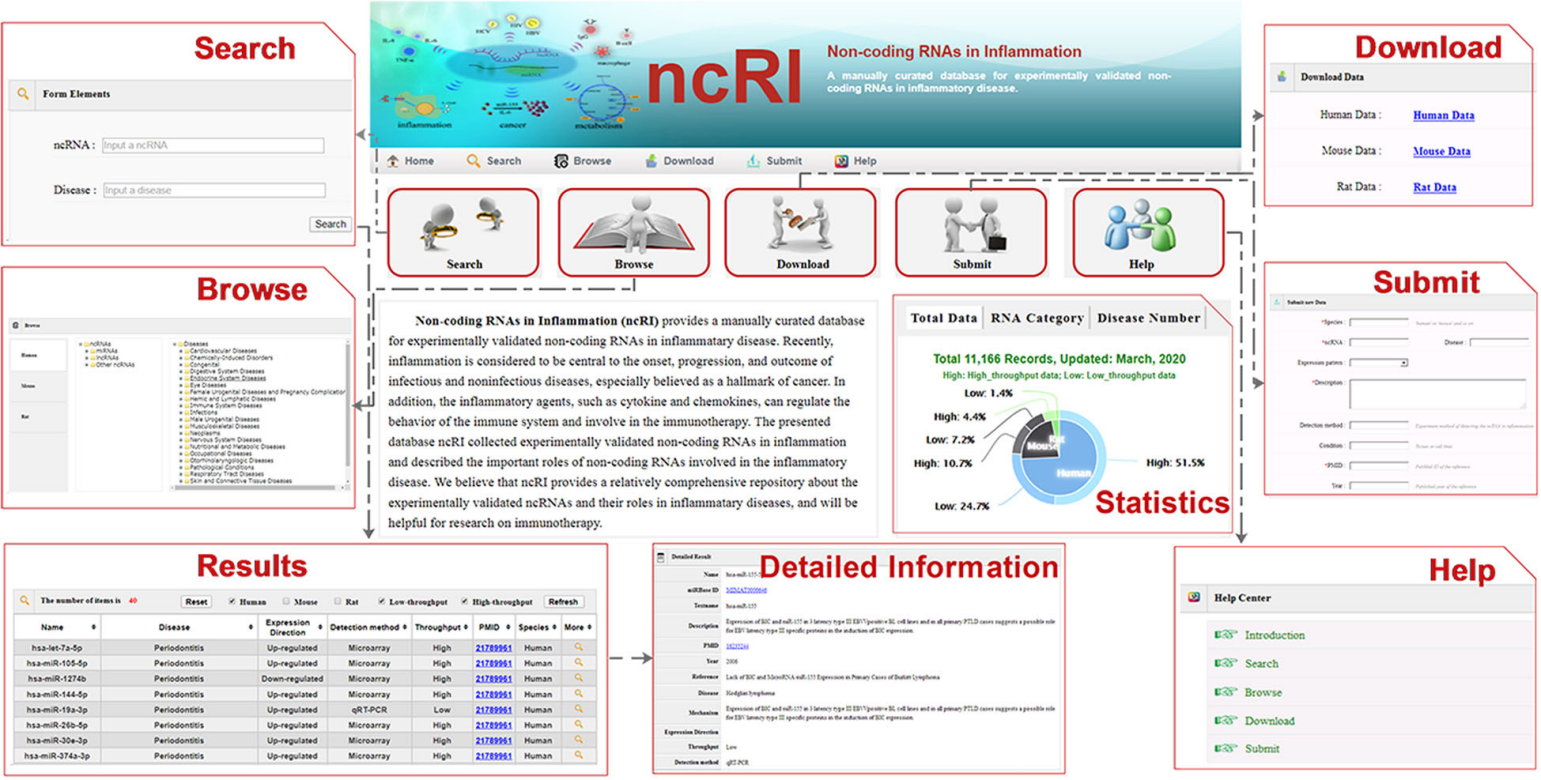
An overview of ncRI. NcRI provides convenient interface for users to access the data

NcRI provides a convenient interface for users to access the data (Fig. 1). The desired ncRNA or disease can be searched by users through ‘Search’ page. Users can input a ncRNA or disease in the textbox. Here, we support fuzzy keyword searching by providing the most relevant matching search. The description about the inputted ncRNA or disease will be returned in the ‘Results’ page. When clicking the ‘more’ button, users can get the detailed information about the entry, including mechanism, detection methods and so on. We provide an option in the ‘Results’ page that allowed users to filter entries by data type (low-throughput or high-throughput experiments) and species (Human, Mouse and Rat). When resorted to the ‘Browse’ page, users can browse all data in the ncRI, categorized by species, ncRNA and disease. We also provide a ‘Submit’ page for users to help us enrich the database. Novel experimentally validated data submitted by users would be reviewed by our committee. We will periodically update the confirmed associations to ncRI. Furthermore, all the curated data in ncRI can be freely downloaded on the ‘Download’ page.

There are several developed databases archiving the disease-associated ncRNAs, such as Human MicroRNA Disease Database (HMDD v3.0) [11], miR2Disease [12], LncRNADisease [13] and Lnc2Cancer [14]. However these databases are not specifically designed for inflammatory diseases. Currently, the ncRI documented about 300 inflammatory diseases, which supplemented over 100 inflammatory diseases, such as gastritis, melioidosis, ophthalmia, prostatitis, pulpitis, to the other known databases. Furthermore, the ncRI database comprehensively collected various ncRNAs related to inflammatory diseases, such as miRNAs, lncRNAs, circRNAs and so on. The mechanism of ncRNAs involved in the diseases was also provided, which will help to understand the pathogenesis of inflammatory diseases. We made a comparison of ncRI and other ncRNA databases in Table 1.

**Table 1:** Comparision of the ncRI and other disease-associated ncRNA databases

	Inflammatory diseases	Expression pattern	Mechanism	Experimental Sample	Mesh ID	miRNA	lncRNA	circRNA
ncRI	√	√	√	√	√	√	√	√
ImmunemiR	√				√	√		
HMDD	√	√				√		
miR2Disease	√	√				√		
LncRNADisease	√	√		√	√		√	√
Lnc2Cancer	√	√	√	√			√	

**Fig. 2:**
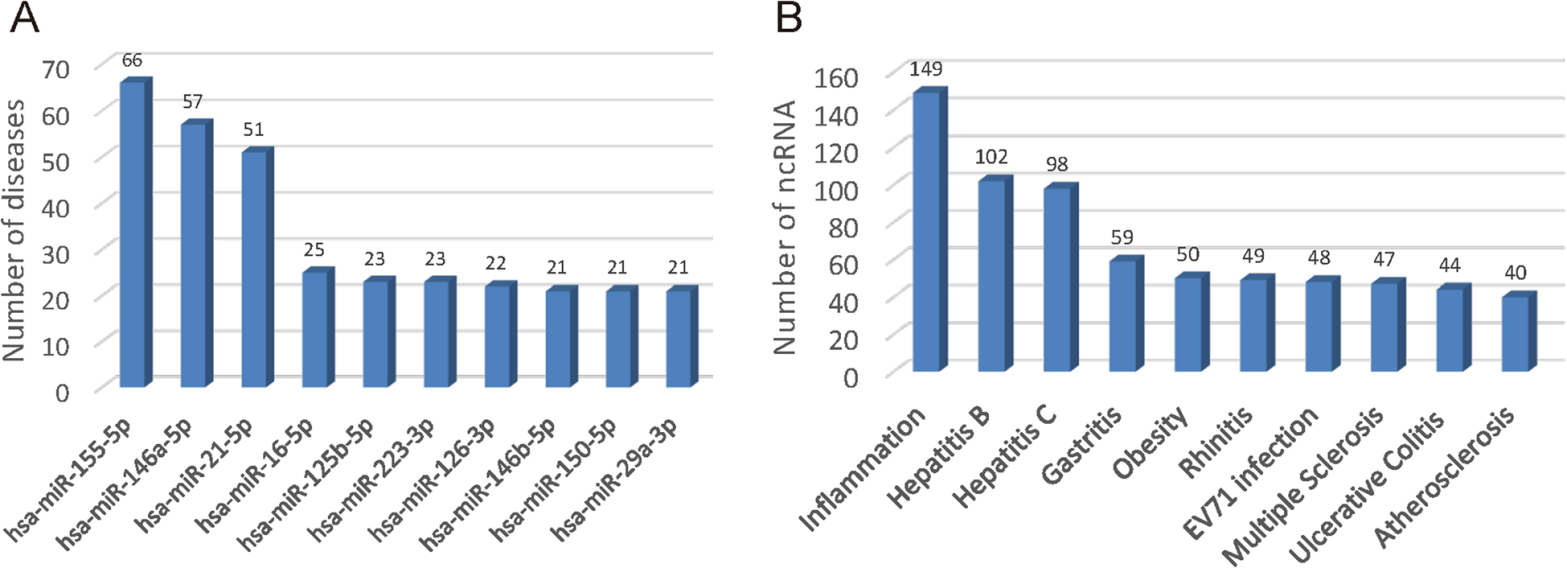
Statistics of the associations of human low throughput data in ncRI. **a** the top ten most inflammatory diseases associated ncRNAs and **b** the top ten most ncRNAs associated inflammatory diseases were presented respectively

In the end, we briefly analyzed the curated data in ncRI. By detecting the total low throughput data in human, we found that miR-155-5p was associated with the most inflammatory diseases, reaching up to 66 diseases, demonstrating the important roles of this miRNA in inflammation (Fig. 2a). Besides the disease inflammation, the hepatitis B and hepatitis C involved most ncRNAs in human, with up to 102 and 98 respectively (Fig. 2b), indicating that the hepatitis was a complex disease that implicated various biological pathways. The top ten most associated ncRNAs and inflammatory diseases of low throughput data in mouse and rat were provided in Figure S1 and Figure S2.

## Conclusions

Inflammation is a necessary process to protect against infection and promote tissue repair, whereas chronic inflammation contributes to the pathogenesis and progression of multiple inflammatory disorders, including inflammatory bowel diseases, arthritis, asthma, diabetes, obesity and cancers. Immunotherapy, which treats diseases by inducing, enhancing, or suppressing an immune or inflammatory response, exhibits a promising therapy for cancers [15]. NcRNAs play important roles in the immune system and emerge as new potential targets for immunotherapy [16, 17]. A comprehensive resource about ncRNAs involved in inflammation will significantly improve our understanding of ncRNAs dysregulation in inflammatory diseases, and promote the development of ncRNA therapeutics.

In this study, we manually retrieved entries about the ncRNAs involved in inflammation from literatures and developed the ncRI database. ncRI provides comprehensive description, mechanism and evidence about ncRNAs involved in the inflammatory processes, including inflammatory diseases. The development and expansion of the ncRI will continue to progress. In the future, the next version will incorporate more comprehensive information and become more powerful.

## Supplementary information

**Additional file 1.**

**Additional file 2.**

## Data Availability

ncRI is freely available at http://www.jianglab.cn/ncRI/.
