# Supplementary figures and images for: ncRI: a manually curated database for experimentally validated non-coding RNAs in inflammation
Source: BMC Genomics. 2020 Jun 1;21:380. doi: 10.1186/s12864-020-06794-6 (PMC7268337; doi:10.1186/s12864-020-06794-6)

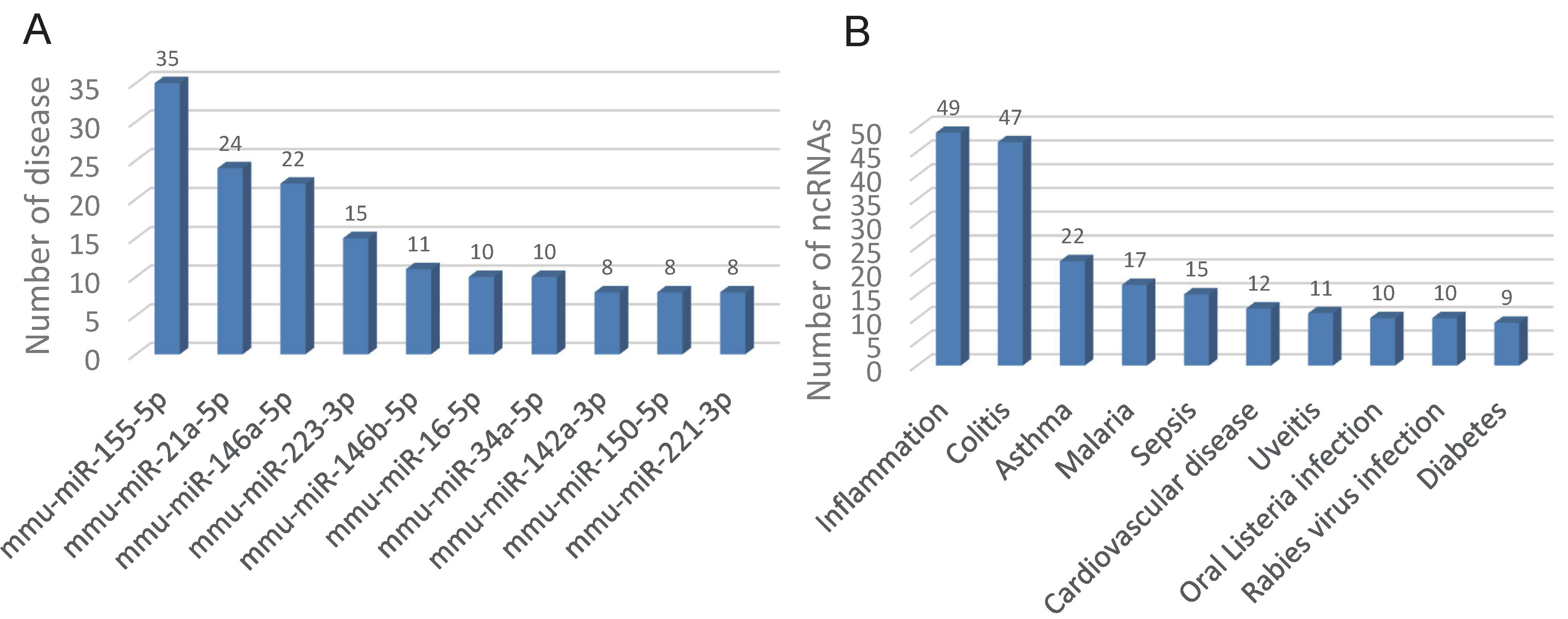

Supplement: Supplementary file 1 — Additional file 1. [file 12864_2020_6794_MOESM1_ESM.png]

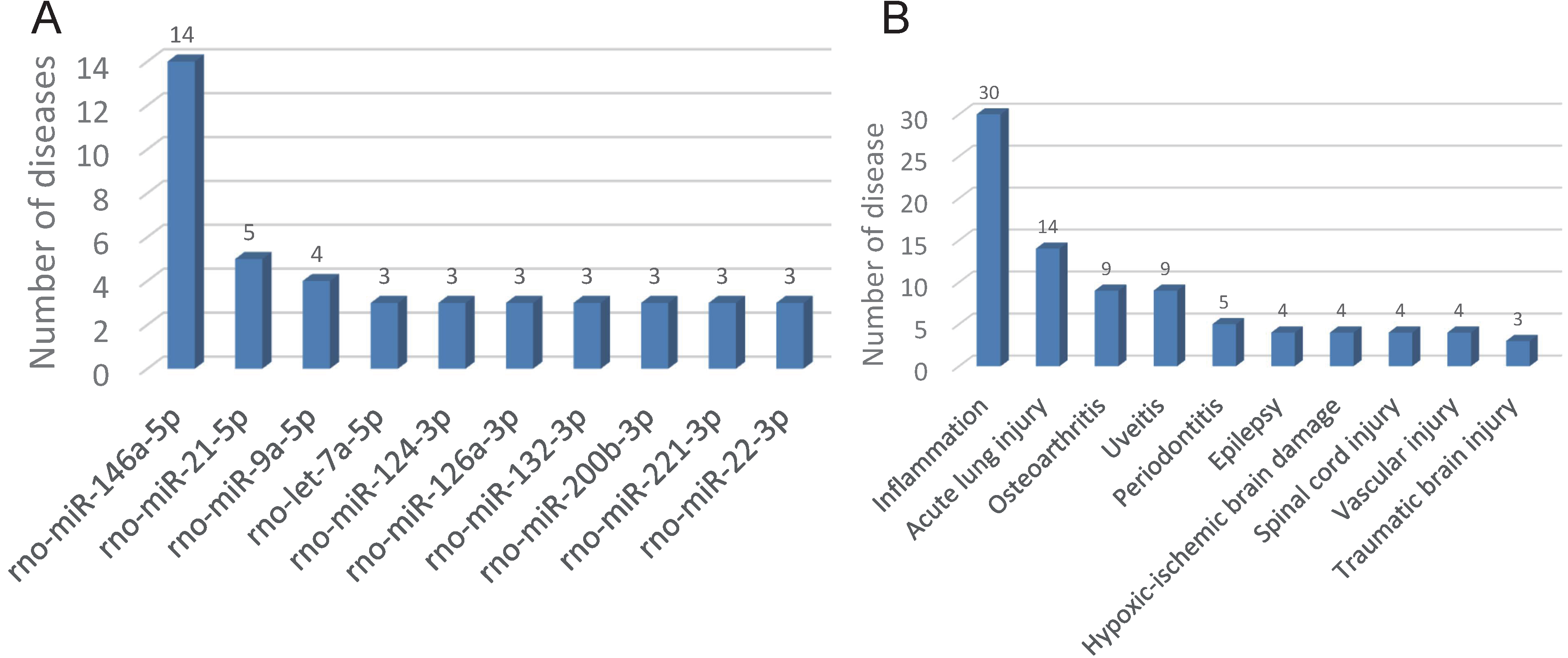

Supplement: Supplementary file 2 — Additional file 2. [file 12864_2020_6794_MOESM2_ESM.png]
